# A cohort study of trends in the prevalence of pregestational diabetes in pregnancy recorded in UK general practice between 1995 and 2012

**DOI:** 10.1136/bmjopen-2015-009494

**Published:** 2016-01-25

**Authors:** Sonia J Coton, Irwin Nazareth, Irene Petersen

**Affiliations:** Research Department of Primary Care and Population Health, University College London, London, UK

**Keywords:** DIABETES & ENDOCRINOLOGY, EPIDEMIOLOGY, PRIMARY CARE

## Abstract

**Objective:**

To describe the characteristics of pregnant women with and without pregestational diabetes and to estimate the prevalence of pregestational diabetes in pregnant women recorded in a UK primary care database.

**Methods:**

The data source for this study is The Health Improvement Network (THIN) primary care database. Pregnant women with and without diabetes aged 16 years and over were identified using diagnostic Read codes and prescriptions for antidiabetics from medical records. Data were examined on: age, body mass index (BMI), social deprivation, smoking, ethnicity and glycaemic control. The prevalence of pregestational diabetes was calculated by diabetes type and calendar year between 1995 and 2012.

**Results:**

Data from 400 434 pregnancies suggests that women with pregestational diabetes were: older (median 29, 32 vs 29 years for type 1, type 2 and without diabetes, respectively), had higher BMI (median 25.0, 30.4 vs 23.9 k/m^2^ for type 1, type 2 and without diabetes, respectively) and were registered with a general practice for longer than pregnant women without diabetes. The prevalence of type 1 diabetes in pregnancy increased from 1.56 to 4.09 per 1000 pregnancies between 1995 and 2015. For type 2 diabetes the increase was from 2.34 to 5.09 per 1000 pregnancies between 1995 and 2008 followed by a more rapid increase to 10.62 per 1000 pregnancies by 2012.

**Conclusions:**

Pregnant women with pregestational diabetes were older, had higher BMI and were registered for longer than women without diabetes. The prevalence of type 1 and type 2 diabetes increased in pregnancy. The prevalence of type 2 diabetes rose more rapidly with a marked increase after 2008.

**Strengths and limitations of this study**

- This study is one of the most comprehensive studies of the prevalence of pregestational diabetes in pregnancy, based on electronic health records in the UK.
- The data source for this study was a large primary care database that is representative of the UK population with over 3 million active patients.
- The study only captures individuals who have been diagnosed with diabetes in primary care as the primary reason for data collection is patient care and management not research.

## Introduction

Diabetes mellitus is a chronic metabolic disease caused by a decrease in the production of insulin or sensitivity to insulin. Type 1 diabetes is caused by the destruction of insulin producing cells in the pancreas and is most commonly diagnosed in childhood. Type 2 diabetes is caused by cells insensitivity to insulin and insufficient production of insulin. Type 2 diabetes is more common among adults, although it is becoming increasingly prevalent in adolescents.^1^

Pregestational diabetes is one of the commonest chronic conditions affecting pregnancy; in the UK 1 in every 250 pregnancies is complicated by pregestational diabetes.^2^ And the prevalence is increasing, in the UK the prevalence of pregestational diabetes increased from 3.1 to 4.7 per 1000 births between 1996–1998 and 2002–2004.^3^

Diabetes in pregnancy is associated with increased risk of pregnancy complications and adverse birth outcomes. Pregnancies affected by pregestational diabetes are at an increased risk of spontaneous abortion, caesarean section, congenital anomalies and perinatal mortality.^3–7^

The current literature on the prevalence of diabetes in pregnancy is based on regional or national samples selected from hospitals, maternity units or small community-based samples.^3^ ^8^ ^9^ We used data from UK primary care records dating back to the 1990s.

The aims of this study were to examine characteristics of pregnant women with and without pregestational diabetes, and to investigate the time trend in the prevalence of pregestational diabetes in pregnancy using data from a large primary care database.

## Methods

### Data source

For this study we used The Health improvement Network (THIN) primary care database, which contains longitudinal anonymised electronic primary care records from 587 general practices, covering approximately 6% of the UK population.^10^ The database contains information on: diagnoses, symptoms, prescriptions, referrals, laboratory tests, basic demographics and social deprivation (recorded by Townsend score). Diagnoses and symptoms are recorded during consultations by practice staff using Read codes, a hierarchical coding system used in UK primary care.^11^

In the UK 98% of the population is registered with a general practitioner (GP) (family physician)^12^ and this is reflected by THIN being broadly representative of the UK population in terms of patient demographics, chronic disease prevalence and death rates.^13^ Practices in the THIN primary care database are located in all nations of the UK, with a slight over-representation of the South of England.^13^

### Study population

We identified pregnant women aged 16 years and over, who were registered with a general practice in THIN and delivered a baby between 1 January 1995 and 31 December 2012. Two data quality measures developed for use within THIN were applied to the data. These data quality measures were; acceptable computer usage (ACU) date and acceptable mortality rate (AMR) date.^14^ ^15^ ACU date is defined as the practice recording on average at least two drug prescriptions and two medical records per patient per year, indicating the practice is using their computing system fully for data recording. AMR date is when the practice has comparable mortality rates to the rest of the UK, in accordance with practice size and demography. Pregnancies were only included after the later of the practice specific ACU and AMR date. Using data after these dates improves the quality.

Pregnant women with pregestational diabetes were identified through diagnoses and prescriptions in their electronic health records. Women who only had one diagnostic code for diabetes or, prescription records alone without a diabetic-specific diagnostic code were excluded from the study as we were not certain of their diabetic status. Women with a first record of diabetes dated after the start of pregnancy were also excluded so that we could be certain we were studying diabetes types 1 and 2, and not gestational diabetes.

We generated an algorithm for classifying diabetes type using different combinations of four variables. The algorithm was discussed in a panel including clinicians. The four variables are: (1) whether a woman had a type-specific diagnostic code; (2) the prescription records; (3) age at first record of diabetes and (4) whether the woman was diagnosed with diabetes prior to entering the practice. The type-specific diagnostic Read code was given the most weight in the type classification algorithm; if a woman had a non-conflicting type-specific Read code then they were initially classified as having that type of diabetes. Prescriptions were then looked at and if a woman had received long-term oral antidiabetics prescriptions with or without insulin, any oral antidiabetics without insulin, or no prescriptions they were classified as having type 2 diabetes. Lastly, for women who had conflicting or no type-specific diagnostic Read codes we considered prescriptions along with age at diagnosis and whether the women were diagnosed prior to registration. In general if a woman had prescriptions for insulin with or without short-term oral antidiabetics, was <35 years old at diagnosis or ≥35 years and diagnosed after registering with a practice they were classified as having type 1 diabetes, and type 2 diabetes otherwise.

After applying the classification algorithm, if there were women still unclassified then a manual review of their entire medical records was undertaken.

### Maternal characteristics

We compared pregnant women with and without diabetes in terms of: age; body mass index (BMI) prior to pregnancy; smoking (coded as never, former or current); Townsend score (coded as 1 least deprived to 5 most deprived); ethnicity (coded as white, black, Asian, mixed or other); blood pressure; and glycated haemoglobin (HbA_1c_) levels prior to pregnancy. We also created a binary variable indicating whether a women was overweight or not; women were classified as being overweight if their BMI measured ≥25 kg/m^2^. All characteristics, apart from maternal age, ethnicity and Townsend score, were captured during the 12 months prior to pregnancy. The record nearest to the start of each pregnancy was taken. Any record of ethnicity prior to pregnancy was selected and the Townsend score for deprivation quintile nearest to the index pregnancy was taken. Maternal age was defined as age at the start of pregnancy.

Townsend score,^16^ is a composite index score of owner occupation, car ownership, overcrowding and unemployment based on a patient's postcode and information from the 2001 census data linked to each patients’ postcode.

### Statistical methods

Characteristics of pregnant women with and without diabetes were compared using median and the IQR for continuous variables or number and per cent for categorical variables calculated for by diabetes type and women without diabetes separately. We randomly selected a single pregnancy per women for these calculations.

We calculated the prevalence of pregestational diabetes by calendar year of delivery and diabetes type for the years 1995–2012 inclusive. For women with multiple eligible pregnancies recorded all pregnancies were included in the prevalence calculations.

All analyses were performed using Stata V.13 (StataCorp, College Station, Texas, USA).

## Results

**Figure 1:**
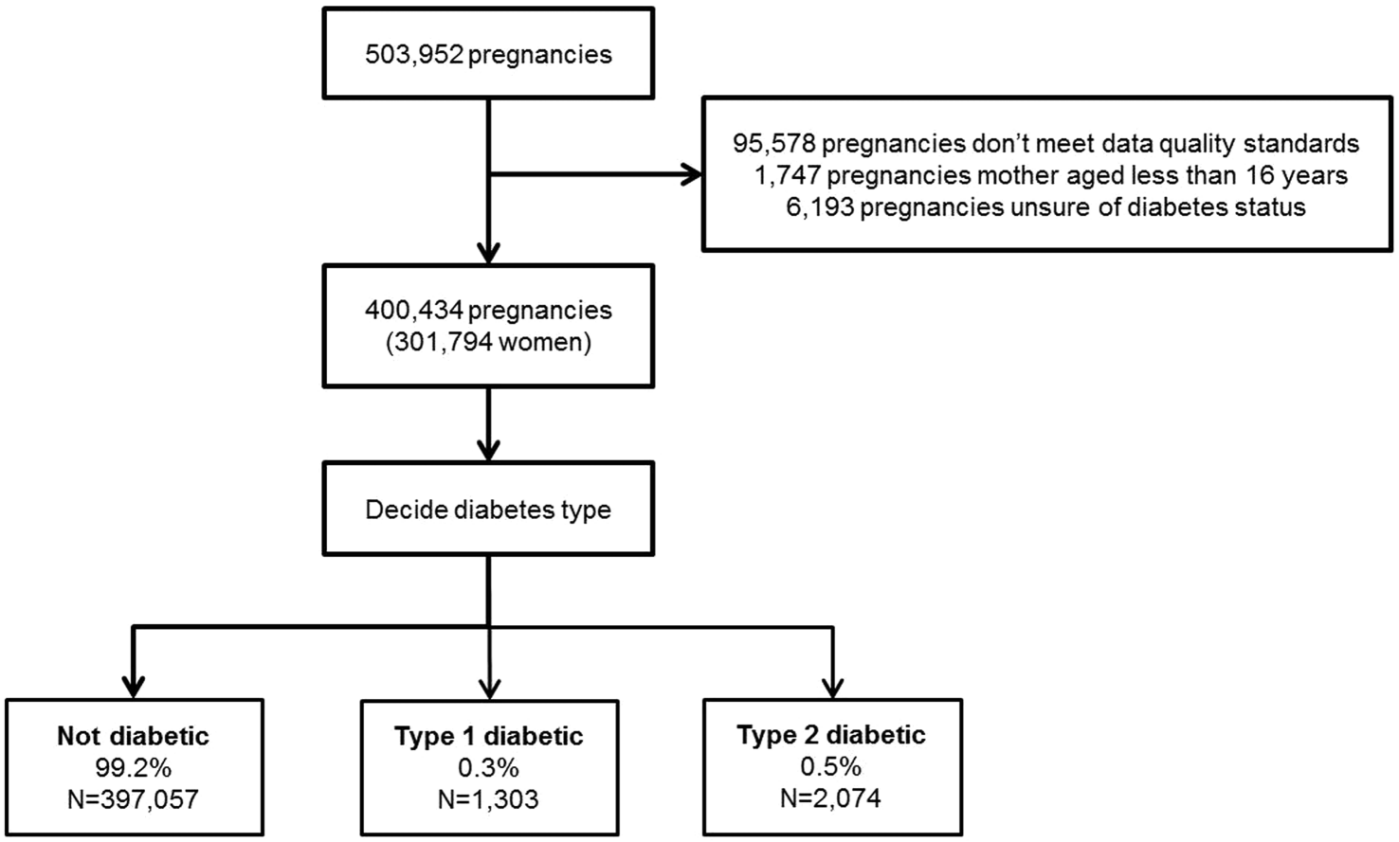
Flow diagram of study cohort development.

We identified 503 952 pregnancies in THIN between 1 January 1995 and 31 December 2012. We removed 95 578 pregnancies as they occurred prior to the practice-specific AMR and ACU date and 1747 pregnancies as they occurred before the mother was 16 years of age. A further 6193 pregnancies were removed because we could not confirm the diabetic status of the mother before the pregnancy began. The final cohort consistent of 400 434 pregnancies to 301 794 women recorded in THIN (figure 1). The majority of women had a single pregnancy (55%) and only 2% of women had four or more pregnancies.

Pregestational diabetes affected 1% of pregnancies from the total cohort: 0.3% (1303/400 434) with type 1 diabetes and 0.5% (2074/400 434) with type 2 diabetes.

For women with more than one pregnancy, a single pregnancy was randomly selected for comparison between women with and without diabetes. Pregestational diabetes affected 0.85% of this partial cohort: 0.3% (999/301 794) with type 1 diabetes and 0.5% (1572/301 794) with type 2 diabetes (table 1).

**Table 1:** Comparison of characteristics of women with and without pregestational diabetes mellitus

	Type 1 diabetes mellitus N=999		Type 2 diabetes mellitus N=1572		No diabetes mellitus N=299 223		
	N	Median (IQR)	N	Median (IQR)	N	Median (IQR)	p Value
Maternal age (years)*	999	29.0 (25.0, 33.0)	1572	32.0 (28.0, 35.0)	299 223	29.0 (25.0, 33.0)	>0.001
BMI (kg/m^2^)†	875	25.0 (22.7, 28.5)	1342	30.4 (25.5, 35.6)	196 368	23.9 (21.4, 27.6)	>0.001
Diastolic blood pressure (mm Hg)†	874	74.0 (69.0, 80.0)	1256	78.0 (70.0, 83.0)	194 217	71.0 (67.0, 80.0)	>0.001
Systolic blood pressure (mm Hg)†	876	119.0 (110.0, 128.0)	1256	120.0 (110.0, 130.0)	194 528	117.0 (110.0, 124.0)	>0.001
HbA_1c_†							
Percentage	557	7.9 (7.0, 9.3)	485	7.0 (6.0, 8.4)	18	5.4 (5.2, 7.1)	>0.001
mmol/moL	557	62.8 (53.0, 78.1)	485	53.0 (42.0, 68.3)	18	35.0 (33.0, 54.1)	
Random plasma glucose (mmol/L)†	179	8.2 (6.0, 11.3)	376	5.7 (4.7, 8.3)	17 779	4.7 (4.3, 5.1)	>0.001
Fasting plasma glucose (mmol/L)†	37	6.7 (5.2, 9.9)	108	6.3 (4.9, 8.1)	3210	4.7 (4.3, 5.0)	>0.001
Length of prior records (years)	999	3.0 (1.0, 6.0)	1572	3.0 (1.0, 7.0)	299 223	2.0 (1.0, 5.0)	>0.001
	**N**	**Per cent (95% CI)**	**N**	**Per cent (95% CI)**	**N**	**Per cent (95% CI)**	
Overweight†							
No	354	35.4 (32.5 to 38.5)	243	15.5 (13.8 to 17.3)	100 340	33.5 (33.4 to 33.7)	>0.001
Yes	521	52.2 (49.0 to 55.2)	1099	69.9 (67.6 to 72.1)	96 028	32.1 (31.9 to 32.3)	
Missing	124	12.4 (10.5 to 14.6)	230	14.6 (13.0 to 16.5)	102 855	34.4 (34.2 to 34.5)	
Smoking status†							
Never	428	42.8 (39.8 to 45.9)	719	45.7 (43.3 to 48.2)	127 379	42.6 (42.4 to 42.8)	>0.001
Former	305	30.5 (27.8 to 33.5)	513	32.6 (30.4 to 35.0)	88 820	29.7 (29.5 to 29.9)	
Current	260	26.0 (23.4 to 28.8)	339	21.6 (19.6 to 23.7)	80 186	26.8 (26.6 to 27.0)	
Missing	6	0.6 (0.3 to 1.3)	1	0.1 (0.01 to 0.5)	2838	0.9 (0.9 to 1.0)	
Ethnic group‡							
White	421	42.1 (39.1 to 45.2)	587	37.3 (35.0 to 39.8)	106 507	35.6 (35.4 to 35.8)	>0.001
Mixed	5	0.5 (0.2 to 1.2)	12	0.8 (0.4 to 1.3)	1234	0.4 (0.4 to 0.4)	
Black	7	0.7 (0.3 to 1.5)	56	3.6 (2.8 to 4.6)	4725	1.6 (1.5 to 1.6)	
Asian	17	1.7 (1.1 to 2.7)	138	8.8 (7.5 to 10.3)	9248	3.1 (3.0 to 3.2)	
Other	9	0.9 (0.5 to 1.7)	26	1.7 (1.1 to 2.4)	3369	1.1 (1.1 to 1.2)	
Missing	540	54.1 (51.0 to 57.1)	753	47.9 (45.4 to 50.4)	174 140	58.2 (58.0 to 58.4)	
Townsend quintile§							
1	223	22.3 (19.8 to 25.0)	251	16.0 (14.2 to 17.9)	65 220	21.6 (21.6 to 21.9)	>0.001
2	195	19.1 (17.2 to 22.1)	263	16.7 (15.0 to 18.7)	55 909	18.6 (18.5 to 18.8)	
3	200	20.0 (17.6 to 22.6)	341	21.7 (19.7 to 23.8)	607 815	20.3 (20.2 to 20.5)	
4	195	19.5 (17.2 to 22.1)	344	21.9 (19.9 to 24.0)	58 600	19.6 (19.4 to 19.7)	
5	143	14.3 (12.3 to 16.6)	280	17.8 (16.0 to 19.8)	44 303	14.8 (14.7 to 14.9)	
Missing	43	4.3 (3.2 to 5.6)	93	5.9 (4.9 to 7.2)	14 376	4.8 (4.7 to 4.9)	

*Age at start of pregnancy.

†Recorded in the 12 months prior to pregnancy.

‡Recorded at any time prior to pregnancy.

§Record nearest to the start of the pregnancy was taken.

BMI, body mass index; HbA_1c_, glycated haemoglobin.

Pregnant women with pregestational diabetes were: older, had higher BMI, were more likely to be overweight, were less likely to smoke and were registered with a general practice for longer prior to pregnancy when compared with pregnant women without diabetes. The median age was 29, 32 and 29 years for pregnant women with type 1 diabetes, type 2 diabetes and without diabetes, respectively. The median BMI was 25.0, 30.4 and 23.9 kg/m^2^ for pregnant women with type 1 diabetes, type 2 diabetes and without diabetes, respectively. Seventy per cent of pregnant women with type 2 diabetes were overweight compared with 52% of pregnant women with type 1 diabetes and 32% of pregnant women without diabetes. The median length of registration prior to pregnancy was 3 years for pregnant women with type 1 and type 2 diabetes and 2 years for pregnant women without diabetes (table 1).

Pregnant women with type 1 diabetes had higher blood glucose concentrations when compared with pregnant women with type 2 diabetes. Median HbA_1c_ concentrations prior to pregnancy were 62.8 mmol/mol (7.9%) for women with type 1 diabetes compared with 53.0 mmol/mol (7.0%) for women with type 2 diabetes (table 1). Pregnant women with type 1 diabetes were also more likely to have blood glucose control tested prior to pregnancy when compared with pregnant women with type 2 diabetes; 56% vs 31% for HbA_1c_, 18% vs 24% for random plasma glucose and 4% vs 7% for fasting plasma glucose.

Pregnant women with type 2 diabetes were more likely to be non-smokers or ex-smokers, and were more socially deprived than pregnant women with type 1 diabetes (table 1). Forty-six per cent of pregnant women with type 2 diabetes were non-smokers, and 33% were ex-smokers at the start of their pregnancy compared with 43% and 31% of pregnant women with type 1 diabetes. Eighteen per cent of pregnant women with type 2 diabetes were in the most socially deprived Townsend quintile compared with 14% of pregnant women with type 1 diabetes (table 1). Diastolic and systolic blood pressure and ethnicity were similar across the three groups, although approximately half of the women did not have their ethnicity recorded.

**Figure 2:**
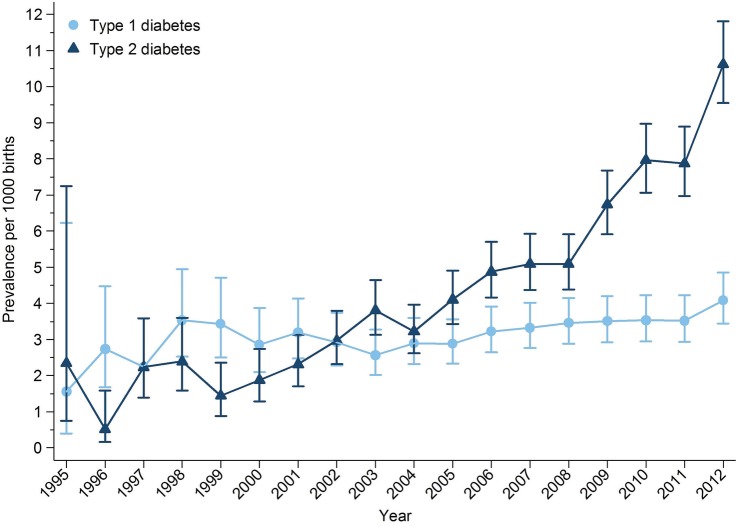
Prevalence of pregestational diabetes mellitus in pregnancy by year and diabetes type.

The prevalence of pregestational type 1 diabetes and type 2 diabetes in pregnancy increased over the study period (figure 2). The prevalence of type 1 diabetes in pregnancy increased from 1.56 to 4.09 per 1000 pregnancies between 1995 and 2012, a 162% increase over 17 years. The prevalence of type 2 diabetes in pregnancy increased from 2.34 to 5.09 per 1000 pregnancies between 1995 and 2008. After 2008 the prevalence of type 2 diabetes in pregnancy increased more rapidly from 6.74 to 10.62 per 1000 pregnancies between 2009 and 2012 (figure 2). Over the study period, the prevalence of type 2 diabetes in pregnancy increased by 354%.

## Discussion

### Summary of main findings

Women with pregestational diabetes were: older, had higher BMI, were more likely to be overweight, and were registered with a general practice longer prior to pregnancy when compared with pregnant women without pregestational diabetes. The prevalence of type 1 and type 2 diabetes in pregnancy increased between 1995 and 2012 with a sharper rise in type 2 diabetes, in particular in the last 5 years of the study period.

### Comparison with existing literature

There are few studies with the primary objective to investigate the prevalence of pregestational diabetes in pregnancy^3^ ^9^ ^17–23^ of these studies only one is based in the UK.^3^ Bell *et al*,^3^ studied the trends in prevalence of pregestational diabetes in pregnancy in maternity units in the North of England between 1996 and 2004, and found comparable prevalence of type 1 diabetes but much lower prevalence of type 2 diabetes than our study. In 2002–2004 they found a prevalence of 3.5 per 1000 births of type 1 and 1.2 per 1000 birth of type 2 diabetes. Of the non-UK-based studies de Andrés *et al*^21^ found in Spain that the prevalence of pregestational diabetes increased from 0.2% in 2001 to 0.27% in 2008. Lawrence *et al*,^9^ in the USA also found that the prevalence of pregestational diabetes more than doubled between 1999 and 2005 from 0.11% to 0.55% equivalent to our findings. Bardenheier *et al*^19^ used state inpatient databases for 19 US states and found pregestational diabetes increased from 0.65 per 100 deliveries to 0.89 per 100 deliveries between 2000 and 2010. The prevalence is comparable to our study in 2000 but the prevalence in the US did not increase as rapidly. Feig *et al*'s^23^ recent study using administrative health claims data for Ontario, Canada found that the prevalence of pregestational diabetes increased from 0.7% in 1996 to 1.5% in 2010, which are comparable to our findings for the same years: 0.3% in 1996 to 1.2% in 2010.

The Confidential Enquiry into Maternal and Child Health (CEMACH) in England, Wales and Northern Ireland reported prevalence of pregestational diabetes between 1 March 2002 and 28 February 2003 as part of a series of findings.^8^ They found a prevalence of type 1 and type 2 diabetes of 2.7 and 1.0 per 1000 births, respectively. Our findings are comparable to CEMACH for the prevalence of type 1 diabetes in pregnancy in 2002 but higher for type 2 diabetes in pregnancy in 2002. The CEMACH enquiry^8^ found pregnant women with type 1 diabetes are different to pregnant women with type 2 diabetes in terms of age, ethnicity and parity. They found pregnant women with type 2 diabetes were older than women with type 1 diabetes; median age 33.5 years compared with 30.0 years, respectively. These results compare favourably to the findings presented in this paper.

Globally there has been an increase in the prevalence of type 2 diabetes among women of child-bearing age. There have been a number of reviews on the prevalence of type 2 diabetes in pregnancy.^24–26^ Lapolla *et al*,^25^ reported type 2 diabetes affecting between 3.2% and 70% of pregnancies globally compared with our finding of 52% across the study period.

### Strengths and limitations

THIN is a large primary care database capturing real-life data from primary care and this was a significant strength of this study. However, THIN was not created for research purposes rather, it is clinical data entry system.

First, there is a large amount of missing data particularly for overweight and ethnicity variables. For pregnant women with type 1 and type 2 diabetes 13% and 16% had missing BMI data compared to 34% missing for pregnant women without diabetes. Whereas for ethnicity approximately half of pregnant women have missing data across the three categories; 54%, 47% and 58% for women with type 1 diabetes, type 2 diabetes and without diabetes, respectively.

A potential limitation of this study is underestimation of diabetes. First, we used diagnostic Read codes, prescriptions and free text entered by GPs to confirm diabetes and we excluded those with only one recording of diabetes and those receiving prescriptions for antidiabetics without a diagnostic code. Second, as many of half those with type 2 diabetes are undiagnosed as symptoms of hyperglycaemia go undetected.^27^ Third, the algorithm used to identify and classify women with type 1 or type 2 diabetes was specific, limiting any false-positive cases of diabetes. Finally, our data is restricted to general practice attenders and women with diabetes who receive their care privately or in specialist clinics would have been missed, contributing to under-reporting of prevalence. Despite these considerations, the study reported higher than expected levels of diabetes then has previously been reported.

### Clinical implications

The increase in both type 1 diabetes and especially type 2 diabetes with a sharp rise in past few years is of special concern to primary care doctors who have to be prepared to work more closely with secondary care on timely management of this problem. There is an established link between diabetes in pregnancy and adverse pregnancy outcomes, including congenital anomalies, perinatal mortality, spontaneous abortion, and delivery by caesarean section.^3^ ^5^ The poorer glycaemic control of women with type 1 diabetes compared with women with type 2 diabetes prior to pregnancy needs particular attention in terms of its risk to the pregnancy and the baby.

It is known that two-thirds of women with pregestational diabetes receive suboptimal preconception care.^8^ In this study we found that half of women with type 1 diabetes and two-thirds of women with type 2 diabetes did not have a HbA_1c_ recorded in general practice in the 12 months prior to pregnancy. With the high levels of problems in primary care, GPs can play a pivotal role in delivering preconception care to reduce the risk of adverse outcomes.^28–30^ This can include both preventive management of all women with diabetes of child-bearing age and more specific management of diabetes during pregnancy. There is a growing need for the development and evaluation of such interventions in primary care.
